# The influence of eating disorders on mothers’ sensitivity and adaptation during feeding: a longitudinal observational study

**DOI:** 10.1186/1471-2393-14-274

**Published:** 2014-08-14

**Authors:** Claire Squires, Christophe Lalanne, Nasha Murday, Vassiliki Simoglou, Laurence Vaivre-Douret

**Affiliations:** Sorbonne Paris Cité, CRPMS, Université Paris-Diderot, EA 3522, Hôpital Cochin-Port-Royal, Paris, France; Département de Recherche Clinique, Hôpital Saint-Louis, Paris, France; CRPMS, EA 3522, Université Paris Diderot, Paris, France; Sorbonne Paris Cité, Université Paris Descartes, Kragujevac, France; Département de Pédiatrie, AP-HP, Paris Centre Hospitalier Port Royal-Cochin, Paris, France; Département de Psychiatrie Infantile, Hôpital Necker-Enfants Malades, Paris, Paris, France; Inserm Unit UMR-SO 669, Université Paris Sud, Paris Descartes, Paris, France; Inserm Unit UMR-SO 669, Université Paris Sud, Paris Descartes, Paris, France

**Keywords:** Eating disorders, Pregnancy, Eating disorder questionnaire, Symptom Check-List, Mother-infant feeding scale, Maternal sensitivity

## Abstract

**Background:**

Parents with past and current eating disorders (ED) have been shown to report troubles nourishing their infants. This could increase the risk of infant feeding problems linked to maternal anxiety and depression. It is not clear how mothers’ eating difficulties before pregnancy and at the time of birth can affect infant’s feeding. We aimed to specify the impact of eating disorders on mothers’ adaptation and sensitivity to their offspring during feeding, by comparing a population of mothers with eating disorders and controls.

**Methods:**

Twenty-eight women agreed to participate in interviews and filmed mother-baby interactions. Pregnant women consulting at an obstetric unit for care follow-up were screened and tested for symptoms of eating disorders with the EDE-Q Questionnaire (Eating Disorders Examination Questionnaire) and the EDE Interview (Eating Disorders Examination Interview). Infant functional troubles and mothers’ sensitivity were investigated through the Symptom Check List. Reciprocal adaptation during feeding with their new-borns was filmed and analysed with the Chatoor Infant Feeding Scale. Before pregnancy, two women suffered from anorexia, three suffered from bulimia, three had binge eating symptoms and two were diagnosed with EDNOS (Eating Disorders Not Otherwise Specified).

**Results:**

Mothers suffering from ED tended to show more difficult interactive patterns in terms of dyadic reciprocity when feeding their babies compared with mothers with no symptoms of eating disorders. In the interviews, other than the behavioural data gathered, ED mothers expressed feeling more dissatisfaction and uneasiness during feeding.

**Conclusions:**

Pregnancy seems to be an useful period for interviewing women on eating disorders, allowing for the design and implementation of prevention programmes based on mothers’ narratives and infant/mother observations and treatment.

## Background

Eating disorders (ED) affect 5 to 7% of women of reproductive age and usually appear to be under-evaluated during the perinatal period [1, 2]. Many studies have investigated eating disorders during adolescence but fewer have been concerned with the perinatal period.

Epidemiological studies rather than clinical surveys have shown that the effect of ED on the course of pregnancy intensifies when ED are more active [1, 3–6]. When eating disorders are active, most studies show that symptoms attenuate, whereas in studies like Blais [7] and Micali [1] they still endure, taking the form of induced vomiting, dissatisfaction with body image, excessive exercise, compensatory dietary restrictions and anxiety. Many women, trusting that pregnancy will help them to overcome ED, consent to eat healthily for the sake of the baby. When ED are residual, pregnancy seems uncomplicated, with importance attached to having a healthy child and a higher level of support for changes in body image.

Women are often reluctant to inform the medical staff about their problems with eating disorders [8] probably because they feel anxious and guilty about harming the foetus. For women suffering from ED, pregnancies are often unplanned and ED pathology has been associated to various perinatal risks such as delayed development, prematurity, hypotrophy, stillbirth, dystocia delivery and postnatal depression. For these reasons, it can be worthwhile to investigate eating disorders during the perinatal period [1, 5, 8–10]. However, eating disorders are not part of the regular obstetric check-ups [4, 11]. Some authors raise the difficulties to detect and diagnose these troubles with appropriate instruments [8, 7, 12, 13].

Eating disorders may also affect infant’s development and health. In fact, parents with past and current eating disorders have been shown to report problems feeding their infants [14, 15]. The nature of the link between parental eating disturbances and infant feeding difficulties can be explained by many determinants, such as genetic influences, children’s temperament and appetite, parental eating psychopathology, affective psychopathology and learnt behaviours [16]. Children of anorexic and bulimic mothers may also show more emotional, conduct, and hyperactivity disorders [17]. It is therefore necessary to study reciprocal influence between mother’s eating disorders and infants’ feeling behaviour [5,7,16].

Since successfully feeding requires recognition of the baby’s needs and an adapted attitude of the caregiver to soothe his/her hungriness, mothers with prior or active eating disorders could find it difficult to accomplish this task. Studies on mother’s attitudes towards the infant during feeding and interaction are discordant. Waugh and Bulik [18] found no significant differences between otherwise similar mothers with and without on behavioural interactions between infant (aged 1–4) and parents during mealtimes, mothers without eating disorders addressed more positive comments to their babies. In other studies, e.g. Stein [19], when compared with controls, mothers who had experienced eating disorders in the post-natal period were more intrusive during their infants’ mealtimes and play and expressed more negative emotions towards their infants during mealtimes but not during play. There are, however, no differences between the groups in their positive expressed emotions.

A controlled style of interaction has been observed in women with eating disorders and offspring who feed with difficulty [19]. There are more conflicts during the meal for mothers with eating disorders. Moreover, mothers with eating disorders feed their babies more irregularly and food is used for non-nutritive purposes. Mothers seem particularly preoccupied by their babies’ weight and appearance, being worried that the baby may be too fat, especially when the baby is a girl [20, 21]. When infants enter early childhood, schemes such as calorie restriction and rigidity in eating rules (no candies or no snacks between meals) begin to appear. Children subjected to these schemes often show a deregulation of eating rhythms and refuse to eat, a disorder characterised by withdrawal and opposing behaviour in communicative exchanges with their mothers. Thus, the child’s growth and development could be affected by conflicts during meals, which have a durable effect by age 10.

However, it is not clear how mothers’ eating disorders before pregnancy and at the time of birth influence when feeding their babies. The mother’s pathological attitudes towards eating, body shape and weight, could have a direct effect on the child and on the way she nurtures him/her, altering the mother’s sensitivity towards the child’s needs and reactions and impinging the quality of the relationship [22]. Micali has asked whether maternal distress and child development and temperament are factors affecting eating behaviours for both mothers and children, concluding that mother’s anxiety and depression could increase feeding difficulties in children and could, in turn, increase distress in both parties over time [23].

Based on this contemporary literature review, we conclude that subjective aspects of reciprocal mother/infant adaptation and mother sensitivity to the infant’s needs and reactions during feeding have not been explored.

### Study objectives

In this study, we aimed to examine mother’s subjective attitude towards their infant and describe parent–child dyads’ interactions during meals (infants’ general characteristics, mothers’ satisfaction, mother’s ease and comfort in feeding, infant regurgitation, satisfaction during meals). We studied a population of women consulting for pregnancy follow-up in an obstetric unit to screen and diagnose different aspects of eating disorders during gestation and to detect possible feeding difficulties with their babies 3 months after delivery.

More precisely, our aims were as follows:

1. To specify recent or chronic symptoms of eating disorders. How do these symptoms evolve during the maternity period? What are the clinical characteristics of eating disorders and their associated factors such as obesity, and their consequences such as somatic problems throughout pregnancy and post-partum depression?
2. To find out if eating disorders have an effect on mother-infant reciprocal adaptation during meals.
3. To collect information about mothers’ attitudes towards feeding practises and establish whether eating disorders influence mothers’ sensitivity during feeding.
4. To increase the awareness of obstetric and child psychiatric staff about eating disorders and encourage prevention during pregnancy and postpartum care.

## Methods

Different tools were used for the assessment of eating disorders, postnatal depression, psychiatric pathology, interactions during meals and mothers’ appreciation of their infants’ functional troubles and maternal care.

### Research Tools

The EDE-Q (Eating Disorders Examination Questionnaire) is a version of the EDE (Eating Disorder Examination) interview, a semi-structured interview diagnosing eating disorders based on DSM-IV [24–26]. The EDE-Q consists of 28 questions in seven sections, divided into five sub-scales (restriction, bulimia, food preoccupations, shape preoccupations, weight preoccupations). Different standards exist for adults [27, 28]. Regarding the pregnancy period, however, we had to adjust some EDE-Q items to the gestational state, for example: “Do you wish to have a flat stomach?” was turned into “Do you wish to be hungry?”

We administered the EDE-Q items during the pregnancy period, but we also investigated the period immediately preceding pregnancy by asking pregnant women to answer the same EDE-Q questions about the period of one or two months before conception. We recognise that this retrospective method is partly a reconstruction.

In a previous study, EDE-Q and EDE were compared in a general population of women aged 18–45 (N = 208) [29]. It was found that EDE-Q scores were higher than EDE scores. In 13 cases, a global EDE-Q score of 2.3 showed maximum validity coefficients when associated to the evaluation of objective bulimic episodes and exercise for the purpose of weight control (sensitivity = 0.83, specificity = 0.96, positive predictive value = 0.56). Based on these findings, we considered an EDE-Q global score of 2.3 as the cut-off distinguishing the two groups of mothers, those with and without eating disorders. Moreover, we studied separately the five different sub-scores of EDE-Q and compared them to Chatoor’s sub-scales scores [30].

The MINI (Mini International Neuropsychiatric Interview) is an instrument assessing the existence of psychiatric pathology. All women were assessed with the MINI in order to exclude those with severe psychiatric disorders [31].

The EPDS (Edinburgh Post-natal Depression Scale) is an instrument consisting of 10 questions that evaluates post-natal depression between 4 to 6 weeks post-partum. All women were tested for depression symptoms after delivery. The prevalence of post-natal depression is usually 10 to 15% in a sample of 859 mothers. The gold standard for post-natal depression is 11 or more [32].

The Chatoor Feeding Scale was developed to diagnose feeding problems during infancy by identifying adaptive and maladaptive behaviours exhibited by both mother and infant. These items are rated on four points and are grouped under five sub-scales: 1) dyadic reciprocity, 2) dyadic conflict, 3) talk and distraction, 4) struggle for control, and 5) maternal non contingency. All dyads were filmed during a meal at a home visit and were separately rated by two independent observers not having previously encountered the dyads [30]. All norms pertaining to the Chatoor Feeding Scale, were applied in our study. Films were analysed separately by two different clinicians not informed of the ED diagnosis.

The Symptom Check List was adapted from Robert-Tissot and Cramer’s [33] grid concerning functional troubles in infancy. From the existing sections, we selected the items evaluating nutrition, sleep, digestion, allergy, care, crying, and mother pleasure. The nutrition section focuses on the mother’s observation of her baby: her nurturing attitudes, number of meals, infant sucking habits (pacifier, sucking fingers), perception of hungriness, mother soothing attitudes. It also outlines the mother’s satisfaction concerning baby’s weight and length, as well as mother satisfaction about the meals. Questions on feeding are both objective and subjective, for example: “In your opinion does your baby eat enough for his/her height and weight?”, “According to you, are the meals agreeable or difficult for you and the baby?”, “Many infants regurgitate, does yours?”).

### Methodology

Pregnant women attending their first consultation at an obstetric unit for follow-up were screened between 14 and 18 amenorrhea weeks of pregnancy for eating disorders with the EDE-Q. They were informed about the research design and asked to sign a consent form to participate, be filmed after delivery and to publish the results. At 20–24 amenorrhea weeks of pregnancy, an EDE interview was used to confirm the previous findings and specify the history of their eating disorders, if any. They were also tested for psychiatric pathology with the MINI. If necessary, dietetic and psychological support was proposed and they were referred to obstetric staff. To maintain contact with the mothers, a visit at the obstetric unit was organised after the baby’s birth.

Two months after delivery, mothers were asked whether they were interested in continuing with the study. In this case, they answered a second EDE-Q post-partum questionnaire and an EPDS questionnaire for post-partum depression and signed a second video film informed consent. Then, they were contacted by phone in order to organise a home visit. During this session a meal was videotaped and analysed with the Chatoor Feeding Scale and an interview with the Symptom Check List was assessed.

Then, we collected various information about pregnancy in the obstetrical file, such as weight and length for BMI, uterine contractions, premature risks, vomiting, hospitalisations, the delivery term, the form of childbirth (vaginal delivery or caesarean section). We also gathered information about the infant’s sex, weight, length and newborn APGAR test, medical problems and feeding modality (bottle, breastfeeding or mixed). All mothers at obstetric unit are asked whether they want to breast-feed or to bottle-feed. A midwife, dedicated to it, can help them to breastfeeding. After delivery, if mothers wish to breastfeed but don’t succeed, they can give mixed food (breast and bottle). When we mention breastfeeding in the article it means at the time of the videotape.

In total, 71 women completed the EDE-Q. Among these respondents, two groups were constituted: one group of 15 women with a global score EDE-Q ≥ 2.3 before pregnancy and another group of 56 with a score < 2.3 preceding gestation. Some women from the two groups decided to withdraw from the study, either at the second EDE interview or after delivery at the moment of the home visit for the filmed interaction. When asked about the reasons for this decision, they replied that they found questions to be uninteresting or that they could not make themselves available, being preoccupied with the new-borns’ care. Among the women withdrawing from the study with an score EDE-Q ≥ 2.3 before pregnancy, one was finally excluded due to schizophrenic disorder but we met her with her baby, one underwent medical termination of her pregnancy owing to foetal malformation, one refused to proceed and two did not answer the phone. The final sample consisted of 10 participants with ED symptoms and 18 without ED symptoms. Our sample is not statistically representative of the usual prevalence of ED symptoms.

Our study fulfils ethical criteria since approved by the Institutional Ethics Committee of the French Public Health System in Paris (Assistance Publique des Hôpitaux de Paris: AOM10_MIN_SQUIRES20091116).

Two written informed consents were asked from participants in this study, one at the first meeting during pregnancy and the second after delivery for the film during mother-baby mealtime. They were informed about the research design, asked to participate, be filmed after delivery and to publish the results. All information concerning parents and infants was anonymously treated and videotapes were unidentified.

### Population

#### Inclusion criteria

Included participants were pregnant women over 18 years of age who agreed to participate in the research and attended pregnancy follow-up consultations. Women were between January 2009 and August 2010 at two maternity hospitals (Cochin Port-Royal Maternity, University Hospitals of Paris and Beauvais Maternity, Aisne, France).

#### Exclusion criteria

Non-French speaking women or those suffering from psychiatric diseases such as schizophrenia or somatic pregnancy pathology (hypertension, foetal malformation, premature delivery with neonatal hospitalisation, etc.) were excluded from the sample.

## Results

We will present the mothers’ characteristics (socio-economic status, BMI, history of eating disorders, psychiatric psychopathology) and the infants’ general characteristics (modality of childbirth, new-born Apgar score, length and weight, hospitalisations) separately. Subsequently, we will examine infants’ functional troubles regarding sleep, nutrition, allergy and caregiving, assessed using the Symptom Check List. Then, we will explore mothers’ postnatal depression and ED symptoms at different stages of the maternity process: antenatal, gestational and postpartum. Finally, we will examine reciprocal mother-infant adaptation and sensitivity to the baby during a meal as shown by analysis of filmed interactions.

### Mothers’ characteristics

The final sample consisted of 28 mothers. Their mean age was 29.4 (Standard Deviation = 4.7). A mother was excluded because of extreme scores at the Feeding Scale and having given no answer to the questionnaires despite the signature of consents. Another was excluded because of an EPDS superior to 25 and appeared to suffer from schizophrenia. She was referred for psychiatric follow up.

Participants were distributed into three socioeconomic status (SES) groups (A = high to C = low). Half of the cases were in the middle SES group. Before pregnancy, two women had suffered from anorexia, three from bulimia, three from binge eating symptoms and two were diagnosed with eating disorders not otherwise specified (EDNOS) according to DSM-IV criteria.

Concerning the BMI before pregnancy, seven women were moderately overweight (BMI above 25), none were obese (BMI above 30). Whereas, three women were very thin (BMI from 16,5 to 18.5), one was treated for anorexia since adolescence but the two others were not in the ED mothers’ group, they described themselves as being naturally thin. It is though noticeable that one of them was depressed in the postpartum period and non very attuned with her baby. Ten women out of 28 had a history of eating disorders (anorexia or bulimia), independent of their actual BMI (t test, p = 0.576).

BMI did not vary depending on the maternity unit (t test, p = 0.692), nor does it vary depending on the mother’s socio-economic status (ANOVA, p = 0.317) (Table 1).

**Table 1:** **Summary of mothers’ and infants’ characteristics**

	Beauvais	Paris	Overall
	N = 13	N = 15	N = 28
Age (years)	29 (24–30)	30 (28.5–33)	29.5 (26.5–32)
BMI (kg/m2)	21.1 (20.7–23.4)	19.9 (18.6–24.7)	20.9 (19.6–24.1)
SES			
*High*	8 (1)	53 (8)	32 (9)
*Intermediate*	69 (9)	40 (6)	54 (15)
*Low*	23 (3)	7 (1)	14 (4)
EDEQ			
*BP*	0.9 (0.9–0.9)	1.8 (1.0–2.5)	1.0 (0.9–2.1)
*DP*	1.3 (0.4–2.2)	1.1 (0.2–1.5)	1.1 (0.3–2.0)
*PP*	0.6 (0.2–2.9)	1.4 (0.6–1.8)	1.2 (0.3–2.5)
EPDS	5.0 (2.5–11.0)	8.0 (6.0–8.5)	6.5 (5.0–8.8)
Chatoor			
*Dyadic reciprocity*	24 (23–32)	29 (27.5–39)	28 (23–33.8)
*Dyadic conflict*	2 (2–4)	2 (0–4)	2 (0–4)
*Talk and distraction*	2 (1–3)	2 (0–4)	2 (1–4)
*Struggle for control*	0 (0–2)	0 (0–1)	0 (0–1)
*Maternal non-contingency*	2 (1–2)	1 (0–4)	2 (0–2.3)
History of eating disorder	31 (4)	40 (6)	36 (10)
Term delivery (weeks)	39 (38.3–40.5)	40.6 (40.3–41.2)	40.5 (39–41.1)
Gender (female)	46 (6)	60 (9)	54 (15)
Two siblings	38 (5)	33 (5)	36 (10)
Weight (g)	3650 (3420–3940)	3330 (3150–3510)	3430 (3207.5–3932.5)
Height (cm)	50 (49–52)	50 (47.5–51)	50 (48–51.3)
Feeding mode			
*Bottle*	62 (8)	33 (5)	46 (13)
*Breast*	31 (4)	60 (9)	46 (13)
*Mixed*	8 (1)	7 (1)	7 (2)

Descriptive statistics are provided as [median (first and third quartile)] for numerical variable and [frequency (count)] for categorical variables. *Abbreviations*: *BMI* body mass index, *SES* socioeconomic status, *BP/DP/PP* before pregnancy/during pregnancy/post-partum.

### Infants’ characteristics

Children of both sexes were equally represented in the two groups of mothers. Concerning siblings, the infant was an only child in 18 cases out of 28, and this was independent of the women’s age.

A t-test showed that babies’ weight did not depend on the feeding modality (46% of the infants were bottle-fed, 46% were breast fed, while 7% were mixed fed; p = 0.157). Babies’ weight also did not depend on a their mothers’ history of eating disorders (p = 0.162). Additionally, infants’ average weight at birth did not significantly differ for those births at under 40 amenorrhea weeks (n = 9, 3.45 ± 0.41 kg), between 40 and 41 weeks (n = 9, 3.39 ± 0.52 kg), or over 41 weeks (n = 10, 3.60 ± 0.35 kg), as confirmed by an ANOVA (p = 0.561).

### Symptom Check-List (SCL)

Scores on SCL are summarised in Table 2.

**Table 2:** **Scores for the Symptom Check-List**

	0	1	2	3	4
Nutrition	35.7 (10)	32.1 (9)	21.4 (6)	7.1 (2)	3.6 (1)
Sleep	17.9 (5)	46.4 (13)	28.6 (8)	3.6 (1)	3.6 (1)
Digestion	14.3 (4)	28.6 (8)	32.1 (9)	14.3 (4)	10.7 (3)
Allergy	35.7 (10)	35.7 (10)	17.9 (5)	7.1 (2)	3.6 (1)
Care	39.3 (11)	17.9 (5)	21.4 (6)	3.6 (1)	17.9 (5)
Crying	35.7 (10)	25.0 (7)	25.0 (7)	7.1 (2)	7.1 (2)
Mother finding					
the baby easy	57.1 (16)	25.0 (7)	14.3 (4)	3.6 (1)	0.0 (0)

Relative frequencies of item scores for the Symptom Check List (counts are given in brackets). Scores range from 0 (satisfying) to 4 (unsatisfying), except for the last item about mother perception (0 = easy to 4 = difficult).

We attributed one of five scores (on a scale from 0 to 4, 0 referring to very good, 1 to good, 2 to sufficient, 3 to difficult, 4 to very difficult), depending on the qualitative and quantitative data we obtained in various domains such as nurturing, sleep, digestion, allergies, care and crying. The mother’s assessment of her infant’s easiness was also taken into account, as she was asked: “Do you think your child is easy, difficult, or very difficult to care for?” with responses rated on the same 0–5 scale.

Scores for the “nurturing” section were relatively independent of the feeding modality (bottle, breast, mixed; χ^2^(8) = 5.65, p = 0.687). Next, we compared EDE-Q scores with items of the Symptom Check List. A positive correlation was found between mothers’ EDE-Q “food preoccupation” item at the beginning of pregnancy and during the postpartum period and the infants’ “nurturing” section on the Symptom Check-List (Spearman r = 0.436, p = 0.023). Obsessive and invasive thoughts about mothers’ nourishment affected the quality of the baby’s nurturing, because these mothers thought that the mealtime was difficult, with the baby not eating enough or regurgitating. Non-parametric tests of trend for the ranks of across ordered groups indicate no significant association between mothers’ eating disorders and infant feeding-related item scores (p > .05 for all scores).

### EPDS

We also tried to ascertain whether maternal depression was correlated with ED symptoms. The mean postnatal depression score was 7.2 (SD = 4.1), with a range of 0 to 15 (maximum obtainable = 30 points). Four mothers had more than point 11 (gold standard), one of them suffered from bulimia; the others were not in the ED group. Concerning suicidal thoughts when presented with the statement “the thought of harming myself has occurred to me” (item10), 25 women scored 0 meaning never, one woman scored 1 meaning hardly ever (with a total EPDS result of 6) and 2 women scored 2 meaning sometimes (with a total EPDS result of 15 for each woman). The EPDS mean scores was not correlated with socioeconomic status (p = 0.071) or with the mother’s history of eating disorders (t-test, p = 0.857).

### EDE-Q

**Figure 1:**
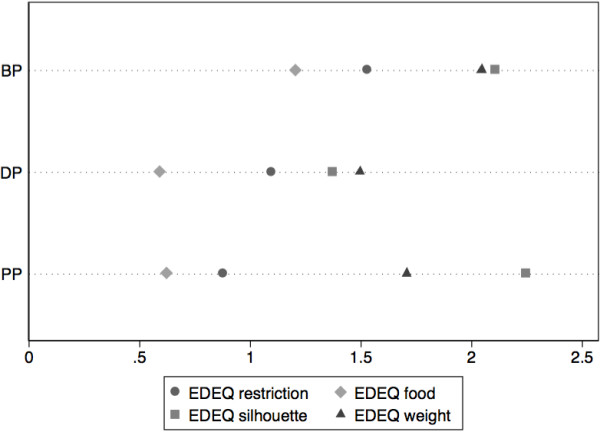
**Scores for the EDE-Q questionnaire.** Mean scale scores for the EDEQ questionnaire before pregnancy (BP), during pregnancy (DP) and in post-partum (PP).

First, mothers having preoccupations with food, shape or weight and showing a restrictive attitude towards food at the beginning of the gestational period, were still concerned by these things after delivery. The joint distribution of scores in the Figure 1 shows a certain consistency in the ratings, with a higher correlation between the scores in early pregnancy and post-partum, compared to the pre-conceptional scores (cf. Spearman correlation matrix, with significance test to 5% adjusted for multiple comparisons). No significant differences were detected between mean scores during the three periods, namely before pregnancy (BP), during pregnancy (DP) and postpartum (PP).Second, no statistical link could be established between mothers’ ED symptoms after birth and infants’ global functional symptoms, because mothers’ postpartum EDE-Q scores did not correlate strongly with infants’ Symptom Check-List global scores (Spearman 0.268), and there was not much variation for SCL items taken individually (score 0 vs. > 0). The distribution of the mean EDE-Q sub-scores for each period is indicated in Figure 1. These scores diminish systematically from the beginning of pregnancy onwards. At the postpartum period, “shape preoccupations” are important. Numerical data (mean and standard deviation) are indicated below.

Third, regarding the evolution of eating disorders before pregnancy and at the beginning of pregnancy, we can state that anorexia or bulimia tended to attenuate, considering that on nine mothers with ED symptoms with a score ≥ 2.3 before pregnancy, only five retain a similar score at the beginning of pregnancy. However, we found evidence of a relapse after birth: For eight mothers with ED symptoms at the beginning pregnancy, the scores EDE-Q remained ≥ 2.3 after delivery.

The forth point is that eating disorders during adolescence were not necessarily reactivated during pregnancy, because eating disorders during adolescence were not related to having an EDE-Q score ≥ 2.3 at the beginning of pregnancy (χ^2^(1) = 3.50, p = 0.061). However, scores before pregnancy highly correlate with ED history at adolescence (χ^2^(1) = 12.28, p < .001).

Moreover, a fifth point shows that post-partum depression is not necessarily a marker of eating disorders. Indeed, EDE-Q scores in the postpartum period correlate weakly with EPDS scores (Spearman r = 0.287, p = 0.155).

Finally, as in many other studies, we found an attenuation of ED symptoms during pregnancy and a reactivation at post-partum, especially for “shape preoccupation”. This finding probably highlights the women’s concern of not recovering a satisfying body image after childbirth.

### *Chatoor*feeding scale

In this section, drawing on Chatoor Feeding Scale, we outline the correlations between mothers’ eating disorder symptoms and their adaptive behaviours with their infants during a meal.

Mean scores on the feeding scale are reported above on Table 1 in the mothers’ characteristics paragraph. The score distribution shows that the dimensions “struggle for control” and “maternal non-contingency” are very asymmetrical, whereas “dyadic conflict” is moderately asymmetrical.

Regarding EDE-Q ≥ 2.3 scores at the beginning of pregnancy, there were no significant differences for the five Chatoor sub-scores (test t, all p > 0,05). Correlations of the five mean sub-scores and EDE-Q in the post-partum appeared non-significant. No correlation could be found between the five sub-scores and sex (t-test), infant weight (Spearman correlation), the hospital (Beauvais or Port-Royal) (t-test) or socioeconomic criteria (ANOVA), so it cannot be stated that these variables are linked to the feeding scale sub-scores.

**Figure 2:**
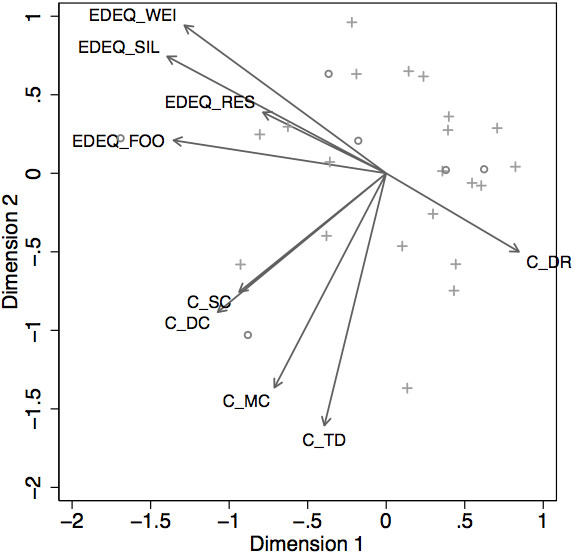
**Biplot of EDE-Q (post-partum) and Chatoor Feeding Scale Scores.** Biplot of EDEQ (post-partum) and Chatoor Scale Scores. Individuals with a total EPDS score above 10 are highlighted with open circles, and a cross marker is used for other subjects. Abbreviations for the EDE-Q questionnaire and Chatoor Feeding Scale: EDEQ_WEI, weight; EDEQ_SIL, silhouette (shape); EDEQ_RES, restriction; EDEQ_FOO, food; C_SC, struggle for control; C_DC, dyadic conflict; C_MC, maternal non contingency; C_TD, talk and distraction; C_DR, dyadic reciprocity. (the smaller the angle is, the more correlated the variables are; variables pointing in opposite directions are correlated negatively).

There was a positive correlation between dyadic conflict and the specific Symptom Check-List item referring to feeding. This finding refers to negative remarks from the mother, infant opposition, distress and anger for both and the overall atmosphere of the meal. This correlation was higher for children feeding from the bottle (Spearman, 0.753) compared with breastfeeding children (0.369), possibly owing to less physical contact.Relations between Chatoor scores and EDE-Q are summarised in Figure 2 as a “biplot” following a principal component analysis of the correlation matrix of scale scores. In the “biplot”, the smaller the angle, the more correlated the variables. Variables pointing in opposite directions are negatively correlated. Principal component analysis (on standardised scores) would lead to retaining two dimensions explaining 66% of the total variance.

The most outstanding result of the “biplot” diagram, is the fact that dyadic reciprocity was negatively correlated with all the EDE-Q sub-scores (for example, r = −0.476 with “shape preoccupation”, r = −0.456 with “food preoccupation”). The more mothers were preoccupied with ED symptoms, the less they were related to their infant and the less they engaged in dyadic interaction during mealtime. Furthermore, it appears that EDE-Q and Chatoor sub-scales, apart from dyadic reciprocity, form two relatively compact sub-sets. “Food preoccupation” appears moderately correlated to “dyadic conflict” (r = 0.654) and “struggle for control” (r = 0.310).

## Discussion

The first aim of this study was to specify the impact of mothers’ eating disorders on the course of pregnancy and post-partum. We were able to confirm the the findings of past research showing that the pregnancy period can have a positive impact on the evolution of eating disorders. Nonetheless, there seems to be a reactivation of the symptoms after childbirth, especially regarding preoccupations on body shape.

Second, concerning postpartum depression, mothers were not more depressed in the postpartum period if they had a history of eating disorders or active symptoms of eating disorders.

Third, concerning mother-infant interaction during feeding, one of the study’s objectives was to study the mothers’ adaptation to their offspring, by comparing a population of mothers diagnosed with eating disorders (based on a gold standard of EDE-Q ≥ 2.3) and control groups. Our main finding was that dyadic reciprocity, reflecting the affective relatedness and quality of mother engagement, was negatively correlated with eating disorders symptoms. Mothers suffering from ED symptoms showed more difficult interactive patterns and less sensitivity when feeding their babies compared with mothers without ED symptoms. Mothers with ED symptoms tended to position their infant less comfortably, make less positive statements about the infant’s feeding skills and food intake and appeared sad or detached, while babies appeared less cheerful, under more stress and did not smile.

Feeding requires a complex conjunction of the baby’s reflex actions and positioning the infant in a favorable position for sucking. The baby needs sufficient comfort and freedom to be active and to regulate his/her intakes or allow himself/herself pauses according to his/her sensations [34]. If feeding is successful in an agreeable atmosphere, it is satisfactory both for the child and the mother. They appear to be in synchrony and experience pleasure during feeding. Consequently, in circumstances of emotional disturbances, many dysregulated interaction patterns can happen during mealtime, such as an uncomfortable position, too much haste to feed or too slowness, the mother experiencing anxiety or the baby sucking slowly.

In our study, the analysis of the video recordings using Chatoor’s Feeding Scales, allowed us to evaluate dyadic interaction and mother’s availability at a behavioural level. Mothers concerned with eating disorders (more preoccupied with “shape concerns” or “food preoccupations”) had less dyadic reciprocity with their infant. They did not position the baby in a way that facilitated visual and bodily reciprocity, they made less positive statements about the baby and his/her intakes and they did not wait for the new-borns to initiate interactions. In reaction, infants appeared stressful and overwhelmed by their emotions.

Interviews with the mothers guided by questions from the Symptom Check List, revealed a more emotional level to some subjective aspects, such as satisfaction and easiness both for the mother and the baby. Mothers with preoccupations about food, experienced more difficulties in feeding their baby; they appeared to be over-concerned with food, complained about more regurgitation and therefore felt that they did not satisfy their babies, which might have led them to become nervous and uneasy during feeding. In a future study, we could draw a clearer distinction between mother’s beliefs, attitudes and affects towards feeding their babies and their interactive behaviours.

As shown by the Symptom Check List analysis, mothers with ED symptom had difficulties recognising the signs of infant hungriness and distinguishing these from a search for contact. When asked “How do you recognise that your child is hungry?”, they replied that he/she cried or shouted. These mothers sometimes fail to grasp that this is a cry for tenderness. They also seemed to have difficulties detecting when the infant was satiated.

As this study focused on infant/mother relationships, we have not considered children’s temperament. Moreover, we have not taken into account mother’s anxiety level in terms of obsessive/compulsive symptoms during pregnancy, and have chosen to exclude from the study one schizophrenic mother. We are aware that this study concerns a limited group of subjects and indicates trends for future research on mother’s sensitivity to children needs. Therefore, further studies on maternal representations can shed more light on the specificities of maternal cathexis of the baby.

As we found that a history of eating disorders tended to reappear in the postpartum period, our study highlights the importance of these troubles’ early detection, in order to enable early interventions for the mother and the infant.

## Conclusions

In our study, we investigated behavioural and subjective preoccupations linked to eating disorders in mothers and infants. We found persistence in eating disorders as measured by the EDE-Q before pregnancy and the postpartum period, though there was relative symptom sedation during pregnancy. All the EDE-Q sub-scales seemed to diminish during pregnancy but increased after delivery, especially concerning “shape preoccupations”. These findings emphasise the importance of treating eating disorders before considering a parental project.

Mothers with eating disorders show more dysfunctional interactive patterns when feeding their babies. These difficulties produce early physical and behavioural negative responses in the baby. The malaise in the relationship can give rise to dysregulated interaction patterns causing disorders in growing children that may endure over time. Thus, infants may develop intense emotions towards food, which can mask the feelings of hunger. Caregiver attitudes affect, therefore, the ability of young children to differentiate between hunger and emotion. In these respects, our study highlights a certain psychic transmission of internal operational patterns around eating.

Pregnancy seems to be an appropriate period for women to be assessed for eating disorders, allowing for the design and implementation of prevention programmes based on mothers’ narratives and infant/mother observations and treatment.

## Abbreviations

EDE-Q: Eating Disorders Examination Questionnaire
EPDS: Edinburgh Postpartum Depression Scale
MINI: Mini International Neuropsychiatric Interview
SCL: Symptom Check-List.
